# Efficient Aggregation of Multiple Classes of Information in Wireless Sensor Networks

**DOI:** 10.3390/s91008083

**Published:** 2009-10-14

**Authors:** Xiaoling Qiu, Haiping Liu, Deshi Li, Jennifer Yick, Dipak Ghosal, Biswanath Mukherjee

**Affiliations:** 1 Department of Computer Science, University of California, Davis, CA, USA; E-Mails: hpliu@ucdavis.edu (H.P.L.); mukherje@cs.ucdavis.edu (B.M.); 2 School of Electronic Information, Wuhan University, China; E-Mail: dsli@whu.edu.cn; 3 Qualcomm Inc., San Diego, CA, USA; E-Mail: jyick@qualcomm.com

**Keywords:** wireless sensor networks, congestion control, fairness, performance analysis

## Abstract

Congestion in a Wireless Sensor Network (WSN) can lead to buffer overflow, resource waste and delay or loss of critical information from the sensors. In this paper, we propose the Priority-based Coverage-aware Congestion Control (*PCC*) algorithm which is distributed, priority-distinct, and fair. *PCC* provides higher priority to packets with event information in which the sink is more interested. *PCC* employs a queue scheduler that can selectively drop any packet in the queue. *PCC* gives fair chance to all sensors to send packets to the sink, irrespective of their specific locations, and therefore enhances the coverage fidelity of the WSN. Based on a detailed simulation analysis, we show that *PCC* can efficiently relieve congestion and significantly improve the system performance based on multiple metrics such as event throughput and coverage fidelity. We generalize *PCC* to address data collection in a WSN in which the sensor nodes have multiple sensing devices and can generate multiple types of information. We propose a *Pricing System* that can under congestion effectively collect different types of data generated by the sensor nodes according to values that are placed on different information by the sink. Simulation analysis show that our *Pricing System* can achieve higher event throughput for packets with higher priority and achieve fairness among different categories. Moreover, given a fixed system capacity, our proposed *Pricing System* can collect more information of the type valued by the sink.

## 1. Introduction

In a Wireless Sensor Network (WSN), sensors cooperate to sense, collect, and report information about the environment to sinks. With the help of multihop wireless communication, a WSN can cover a large area without the infrastructure or a backbone wired network. However, congestion can exist inside a WSN due to the following inherent characteristics. First, in a multihop WSN, resources are limited. Typical sensors have limited battery power, memory, and computing capability. In addition, sensors also need to compete for shared resources inside the WSN, such as the shared wireless channel with neighboring nodes and common paths to sinks. Second, majority of the time, the topology of a WSN is not completely under control. As a result, a lot of traffic might contend for the same links or nodes that can become the bottlenecks of the whole network. This imbalance of network traffic due to the network topology can cause severe congestion in bottleneck nodes and/or links. Third, sensors that detect an important event usually increase the data generation rate to accurately alarm the sinks in time. For example, sensors used for monitoring temperature in a forest will generate a large number of alert packets in a short period of time when they detect fires. Fourth, some new applications, such as patient health monitoring [1] and image sensing [2], require high throughput and low delay, which can further aggravate the congestion inside a WSN. Therefore, congestion control is necessary and inevitable in the WSN. In the absence of congestion control, WSNs can suffer from packet loss due to buffer overflows and inefficient utilization of critical resources such as shared wireless channel capacity and sensor battery power.

Existing proposals to address congestion control in WSNs are either hop-by-hop data rate control or source rate limiting mechanisms. In this paper, we propose a Priority-based Coverage-aware Congestion Control (*PCC*) mechanism in Section 2.. *PCC* operates at the network and MAC layers. It is a distributed method that avoids aggregating network information in the sink and therefore does not require complicated and expensive communication among nodes [3].

**Figure 1.**
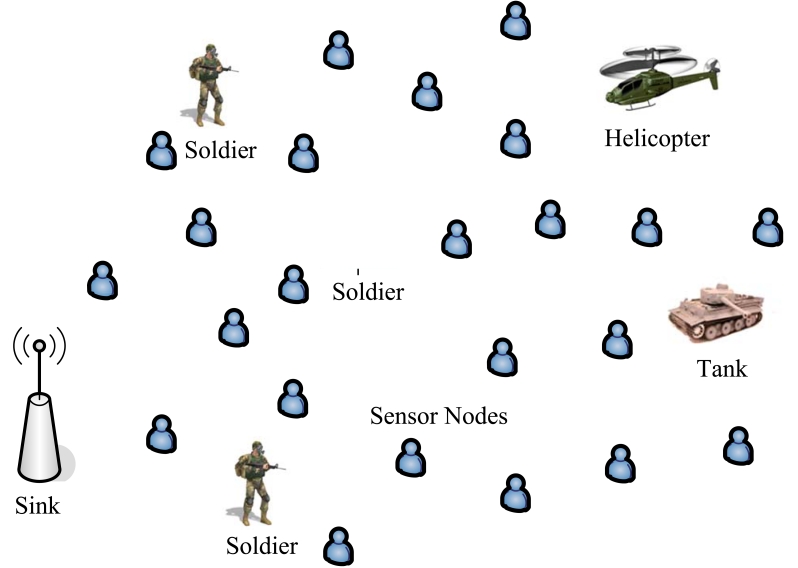
Collection of multiple classes of information in a WSN.

For advanced WSN applications, we expect to collect multiple categories of information from sensor nodes. For example, from an under-water sensor network, we may collect data about the temperature, the degree of ambient light, the pollution level, and other relevant parameters. The sink can request and store different monitored information from the sensors for each data collection cycle. It is much more efficient and economical than using separate overlapping sensor networks to gather different information. Currently, sensor nodes such as the Mote [4] has the capability to gather all the information. The Mote can be equipped with different kinds of sensor interfaces in the circuit board; Arch Rock's EPIC Mote has integrated temperature, light and humidity sensors [5]. However, multiple categories of information contend for the limited network resource to send data from sensors to the sink. Managing the sensors to cooperate and send multiple classes of data fairly and efficiently is a challenging problem, especially when the network is congested. The sensors could ignore the difference between data in the application-layer and send them to the sink with the same weight. However, different data have different value to the sink. For example, in the military application as in Figure 1, the sensor network can collect real-time battlefield information to identify an infantry, a tank, or a helicopter. However, different enemy units pose different level of hazard. The information about hostile helicopters and tanks are important and urgent since they may be more dangerous than infantries. On the other hand, it is also not advisable to assign very high priority to only one type of data. For example, although the data pertaining to hostile tanks are important, it is also important to collect some information regarding infantries. It may be disastrous to utilize all sensor network resources to locate hostile tanks at the cost of ignoring other enemy units.

Consequently, a WSN should be able to allocate network resource to a specific kind of data according to the “price” the sink places on the type of data. Hence, resource consumption depends on the following factors: (1) properties of the events, such as priority, location, and frequency; (2) properties of the sensor network, such as topology and link quality, and (3) other categories of events.

In Section 2., we provide a mechanism to estimate the success probability for transmitting a data packet from a sensor to the sink. The success probability is a good metric for resource consumption and includes both bandwidth and buffer. Based on the method in Section 2., we generalize our scheme to efficiently and fairly collect different categories of information when the WSN is congested. This is presented in Section 3.. The following are the key contributions of this paper:

1. In a WSN, packets with information of the desired event (*Event* packets), such as fires in the forest, are more important and urgent than those without event information (*Non-Event* packets). (Note that *Non-Event* packets are inevitable since sensor nodes need to contact with the sink periodically to notify that they are alive.) Therefore, in *PCC* we distinguish them with different priority thereby providing different throughput and dropping probability.
2. When congestion occurs, packets from nodes far from the sink have a smaller chance to reach the destination than those from the nodes close to the sink [6]. Without any control, the WSN can only collect the information from the nodes near the sinks. Therefore, in *PCC*, we assign packets an index to store the probability of a packet successfully reaching any node along its path to the sink. Then *PCC* can dynamically adjust its dropping probability during congestion, to guarantee fairness for all nodes and coverage fidelity of the whole network.
3. In a large WSN, wireless link quality changes according to multiple factors, such as obstacles between transmitter and receiver, multiple-path transmissions, and interference among neighbor links. In *PCC*, we consider the influence of link quality as an important parameter to indicate network resource utilization and the successful probability of transmissions.
4. We make use of cumulative survival probability of a packet reaching a node along its path to the sink and the priority of different event information to design a mechanism to efficiently and fairly collect different categories of information in a single WSN, called *Pricing System*.

The remainder of this paper is organized as follows. In Section 2., we present the details of the *PCC* mechanism, describe the design objectives, and present the simulation results. In Section 3., we propose a generalized pricing based scheme to efficiently collect multiple categories of information using one WSN. Related research is discussed in Section 4.. Finally, we conclude in Section 5..

## 2. Priority-based Coverage-aware Congestion Control (PCC)

### 2.1. System Model and Design Considerations

**Figure 2.**
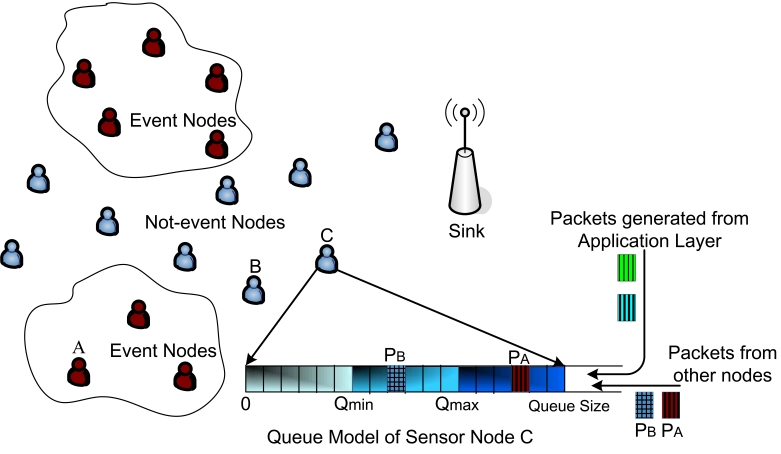
The overall system model.

We design our congestion control mechanism based on the system model shown in Figure 2. We consider a WSN with *N* sensor nodes that act both as source nodes as well as routers to forward packets through a multihop network to the sink. Each sensor node has a fixed size buffer to store packets, which is shown for node *C* in Figure 2. The buffer of node *C* contains packets generated by itself and packets from other sensors, like packet *P_A_* from node *A* and packet *P_B_* from node *B*.

Under normal condition of the physical attribute monitored by the WSN, nodes generates *Non-Event* packets at a constant rate of *r* packets per second (pkts/sec) which are forwarded towards the sink. Upon sensing an event, sensor nodes generate *Event* packets at higher rate, *k* × *r* pkts/sec where *k* ≥ 1, to report the information to the sink. A one-bit field in the packet header is used to identify *Event* packets. The intermediate nodes can use this bit to route packets with different priority.

Based on the above system model, the goal is to find a novel mechanism to efficiently collect information generated by the nodes in the WSN. Before discussing the details of our approach, we first explain the objectives, the design challenges and the corresponding solutions to the challenges.

**Figure 3.**
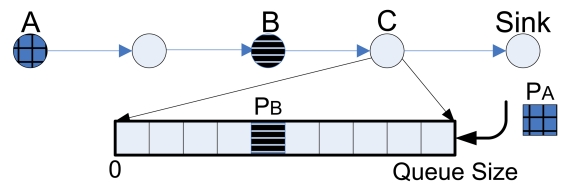
Queue scheduler that allows dropping intermediate packets.

1. High *Event* Packets Throughput: In a WSN with both *Event* and *Non-Event* packets, it is important to ensure that *Event* packet throughput is high. In addition, since *Event* packet generation rate is normally higher than that of *Non-Event* packets, congestion may occur when events are detected by different sensor nodes simultaneously. Therefore, our mechanism should first guarantee high *Event* packet throughput when nodes are congested, to make sure that emergency information, like fire in the forest, is reported to the sink correctly and in a timely manner. We set up two thresholds in the sensor node queue to drop *Event* and *Non-Event* packets to give the former higher priority. To the best of our knowledge, few papers differentiate *Event* and *Non-Event* packets in WSNs with the exception of Event-to-Sink Reliable Transport (ESRT) [7]. Note that our work is different from ESRT, which is implemented in the transport layer and is an end-to-end congestion control method. *PCC*, on the other hand, is distributed and is based on network layer queue scheduling and MAC layer information feedback, as will be discussed in the following sections.
2. Coverage Fidelity: As we explained in Section 1., packet throughput from a specific sensor node drastically decreases when packets traverse multiple hops to the sink. Therefore, packets generated by nodes nearby the sink have much higher probability of reaching the sink than those generated by nodes far away from the sinks. This leads to a spatial bias in the information collected in a multihop WSN. However, it is crucial to achieve coverage fidelity in a WSN because each monitoring area is usually equally important or remote areas are even more important since they are more difficult to be monitored by direct methods. Unlike other proposed methods, we consider the fairness among different areas at the application layer. Our proposed mechanism ensures that the sink receives equal number of packets with the same priority from all the sensor nodes in the sensor network. In IFRC [8], authors describe MAC layer fairness. However, MAC layer fairness does not ensure application layer fairness since the sink is biased to receive packets from nodes that are near it.
3. Flexible Queue Scheduler: Most queue schedulers drop packets from the tail rather than any position in the queue. But tail-dropping does not work well in our new scheme. For instance, if the queue in a sensor node is near fully occupied and dominated with *Non-Event* packets, when an *Event* packet arrives, it is better to drop *Non-Event* packets because *Event* packets are more important. To address *Coverage Fidelity*, we can consider the scenario in Figure 3, where node *B* is closer to node *C* than node *A*, and the sink is at the rightmost end. If node *A* generates packet *P_A_* and node *B* generates packet *P_B_* simultaneously, *P_B_* will normally arrive at node *C* earlier. When *P_A_* arrives at node *C* whose queue is highly utilized, *P_A_* may be dropped while *P_B_* remains in the queue. This results in unfairness to different sensors. To mitigate this our proposed method checks the status of all packets in the queue and selectively drops packets according to an optimization algorithm, which will be introduced in the next section. With the help of a list of pointers to packets in the queue, it is feasible to drop intermediate packets with much lower complexity than expected.
4. Resource Efficiency: Another important concern in a WSN is resource efficiency since sensor nodes usually have limited power and channel bandwidth [9, 10]. In a WSN, packets from sensors far from the sink normally consume more network resource than those from nodes nearby the sink. In our proposed method, we give preference to maintain packets from remote nodes since those packets have consumed more network resource and have lower probability to reach the sink when the intermediate relaying node experiences severe congestion. Therefore, when the same information is collected from different sensors in the network, our mechanism can efficiently utilize network resources by reducing the average number of point-to-point transmissions.
5. MAC/PHY Link Quality: In a multihop WSN, the interference among neighboring links can severely reduce the transmission opportunities in MAC layer. In addition, in a WSN, wireless link qualities, such as noise and channel fading, are quite distinct according to locations, obstacles, etc. The link condition at MAC/PHY layer can also influence the success probabilities that packets reach the sink. In respect to resource efficiency, packets traveling through low quality links consume higher system resource since they require more re-transmission due to MAC collisions or more transmission time due to lower PHY layer transmission rate. Therefore, our mechanism will give packets traveling though poor quality links from remote nodes higher probability to reach the sink.

### 2.2. Protocol and Algorithm Design of PCC

*PCC* is a distributed protocol. First, a distributed protocol is more robust to node or link failures than a centralized protocol. Second, a distributed protocol does not have to collect global information and distribute centrally determined control information, which may introduce large overheads that are not acceptable in a WSN. Third, the distributed algorithm is more scalable in large WSNs.

**Figure 4.**
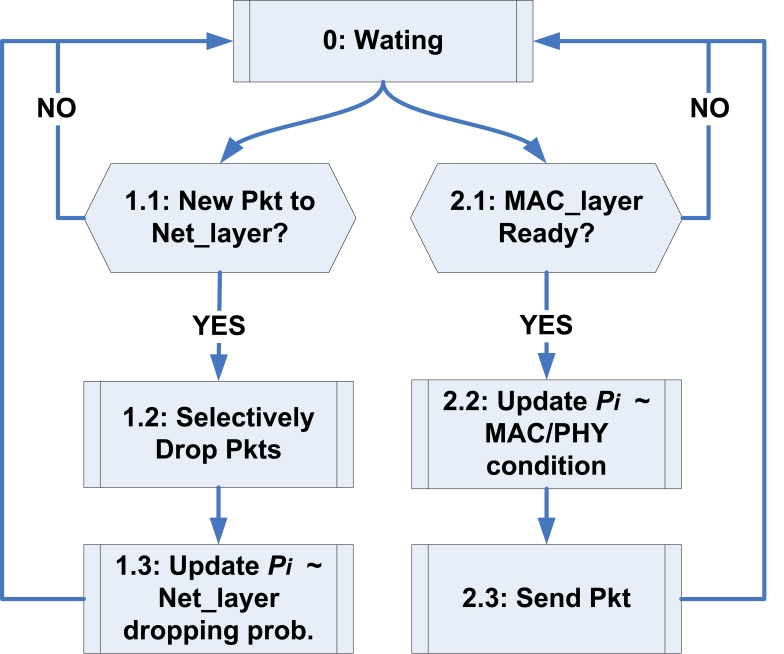
A block diagram illustrating the overall structure of *PCC*.

The protocol structure of *PCC* is shown in Figure 4. It operates both at the network and the MAC layers, which are shown in the left and the right branches, respectively. As in the left branch, when new packets arrive (from the application layer of the node or from other nodes as in Figure 2) into the network queue (Part *1.1 in PCC*), we selectively drop packets in the queue during congestion according to an optimization algorithm (Part *1.2* in *PCC*), introduced in the next subsection. Since our protocol is distributed, each packet *i* has an additional field in the header, *P_i_*, to store the cumulative survival probability of the packet along the path. Therefore, at a sensor node when a packet chosen to be dropped, we need to update the *P_i_* for all the remaining packets in the queue. Furthermore, as in the right branch of Figure 4, when the MAC layer is ready to transmit a packet (Part *2.1* in *PCC*), we need to update *P_i_* of this packet with the probability that the packet will survive in the MAC/PHY link transmission from the node (Part *2.2* in *PCC*), and the packet is then send it to the next hop (Part *2.3* in *PCC*).

When a sensor node *A* generates a packet *i*, we initialize *P_i_* = 1. Along the path from node *A* to the sink, all relaying nodes including *A* updates *P_i_* based on the packet dropping probability in network layer and link layer transmission error and loss in the MAC/PHY layer. The cumulative survival probability of a packet reaching any node in the network is used to determine the dropping probability of the packet in the node.

#### Queue Handler

As we explained in Section 2.1., *PCC* supports two priority classes of packets, *Event* and *Non-Event* packets. In any node in the network, suppose that the total buffer size is *Q* and in the queue there are *N_E_ Event* packets and *N_N_ Non-Event* packets with the total number of packets *N* = *N_E_* + *N_N_*. We set up two thresholds, *Q_min_* and *Q_max_* as shown in Figure 2, for handling different kinds of packets and apply the following logic.

1. 0 ≤ *N* ≤ *Q_min_*: buffer all incoming packets.
2. *Q_min_* < *N* < *Q_max_*: begin dropping *Non-Event* packets while keeping all *Event* packets. The dropping rate is selected such that the average number of *Non-Event* packets *N_N_¯* = *F_N_*(*N*).
3. *Q_max_* ≤ *N* ≤ *Q*: drop all *Non-Event* packets and begin to drop some *Event* packets. The dropping rate is selected such that the average number of *Event* packets *N_E_¯* = *F_E_*(*N*).

Function *F_N_*(*N*) and *F_E_*(*N*) will be discussed later on.

As we discussed in Section 2.1., the coverage fidelity and high *Event* packet throughput are important objectives. Therefore, we cannot randomly select packets in the queue to drop. In order to achieve coverage fidelity we need to give fair chance to all packets from different sensor nodes in the network to reach the sink. In other words, assuming that the accepting probability of a packet *i* is *k_i_* (i.e., (1 − *k_i_*) is the dropping probability), we would like to ensure that ∀*_i_*, *j*, *P_i_* × *k_i_* = *P_j_* × *k_j_*. In this case, we can guarantee that packets from different nodes have the same or similar probability to reach the sink. Therefore, our scheme is to find *K_E_* = [*k*_1_, *k*_2_, …, *k_NE_*] and *K_N_* = [*k*_1_, *k*_2_, …, *k_NN_*] under the following constraints:

(1) $$\sum_{i\in N_{E}}k_{i}=F_{E}(N)$$

(2) $$\sum_{j\in N_{N}}k_{j}=F_{N}(N)$$

(3) $$\forall i,j\in N_{E}P_{i}\times k_{i}=P_{j}\times k_{j}$$

(4) $$\forall i,j\in N_{N}P_{i}\times k_{i}=P_{j}\times k_{j}$$

However, solving the above problem can result in conflicts. For example, consider three (Event) packets with *P*_1_ = 0.1, *P*_2_ = 0.25 and *P*_3_ = 0.5, and *F_E_*(3) = 2. Solving the above problem yields *K_E_* = [1.25,0.5,0.25]. Note that *k_i_* is the accepting probability within [0, 1], so *k*_1_ is not an acceptable probability measure. In other words, it is impossible to strictly guarantee the constraints (3) and (4). To resolve this, we borrow another popular fairness metric, *Jain's Fairness Index*([11]) $\frac{{\left(\sum x_{i}\right)}^{2}}{\left(n*\sum x_{i}^{2}\right)}$, to give a relatively fair opportunity to packets. The optimization problem for both *Event* and *Non-Event* packets can be stated as follows

(5) $${\text{maximize}}_{\left\{K\right\}}\frac{{\left(\sum P_{i}\times k_{i}\right)}^{2}}{N_{P}\times \sum {\left(P_{i}\times k_{i}\right)}^{2}}$$

such that

(6) $$\sum_{i\in N_{P}}k_{i}=F(N)$$

(7) $$\forall i\in N_{P}k_{i}\in [0,1]$$

where *K* is the decision variable and *K*, *N_P_*, *F*(*N*) could be either *K_E_*, *N_E_*, *F_E_*(*N*) or *K_N_*, *N_N_*, *F_N_*(*N*) corresponding to *Event* and *Non-Event* packets, respectively.

It is difficult to implement the quadratic optimization problem in Equation (5) with limited resources in the sensor nodes. Therefore, a simpler algorithm is required. Note that our initial objectives are Equations (3) and (4), so for both *Event* and *Non-Event* packets, the objective can be restated as

(8) $$P_{1}\times k_{1}=P_{2}\times k_{2}=\ldots =P_{N_{P}}\times k_{N_{P}}=c$$

(9) $$\Rightarrow \forall i,k_{i}=\frac{c}{P_{i}}$$

(10) $$\Rightarrow \sum_{i}\frac{c}{P_{i}}=F(N)(∵\sum_{i}k_{i}=F(N))$$

(11) $$\Rightarrow c=\frac{F(N)}{\sum_{i}\frac{1}{P_{i}}}$$

(12) $$\Rightarrow \forall i,k_{i}=\frac{c}{P_{i}}=\frac{F(N)}{\sum_{i}\frac{1}{P_{i}}\times P_{i}}$$

In this solution, if *k_i_* > 1 as discussed above, packet *i* should be kept in the queue, and therefore *k_i_* ⇐ 1. However, this change influences the accepting probabilities of other packets, which need corresponding updates given *k_i_* = 1. The details of how this done is shown in Algorithm 1, which finds the solution for objective in Equation (5).

In Algorithm 1, there are two loops inside the *while* statement, each of which has a complexity of *O*(*N*). The worst case for each execution of the *while* loop is that we separate one *P_i_* from *P⃗* in each iteration with complexity *O*(*N*). Therefore, overall computation complexity of Algorithm 1 is *N* × (2*N*) = 2*N*^2^ which is *O*(*N*^2^).

The solution of this optimization problem, *K_E_* and *K_N_*, gives the accepting probability of each packet in the queue. This can be used by the node to drop packets when the node is congested while maintaining coverage fidelity by giving each packet a fair chance to remain in the queue. After the selection and dropping process, the *P_i_* of each packet *i* is updated to *P_i_* = *P_i_* × *k_i_* for all remaining packets since they experience dropping one more time (Part *1.3* in *PCC*).

**Figure 5.**
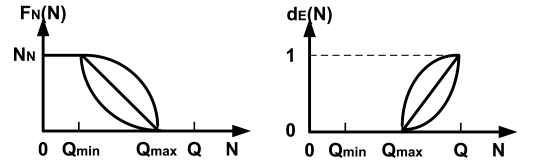
Candidate *F_N_*(*N*) and *d_E_*(*N*) functions.

*F_E_*(*N*) and *F_N_*(*N*) are accepting functions for *Event* and *Non-Event* packets, respectively. In a tail-dropping scheme, when a new packet arrives, the accepting function is (*N* + 1) − *f_drop_*(*N*) where *f_drop_*(*N*) is the dropping probability of the new packet given *N* packets in the queue. In our system, since the queue handler could drop any number of packets, *PCC* can implement different kinds of *F_E_*(*N*) and *F_N_*(*N*) functions. Note that the basic purpose of *F_E_*(*N*) and *F_N_*(*N*) is to reduce the congestion as a result the dropping probability monotonically increases with the number of packets in the queue. In general, *F_N_*(*N*) can be a linear, convex, or concave function within [*Q_min_*, *Q_max_*] through two fixed points, (*Q_min_*, *N_N_*) and (*Q_max_*, 0) as shown in the left part of Figure 5. Clearly, with the convex function, packets are dropped very aggressively resulting in lower buffer utilization, while the concave function is more conservative and will result in higher buffer utilization. The linear function is between the convex and concave functions.

**Algorithm 1** Optimization AlgorithmInput: *P⃗*, *F*(*N*)Output: *K*1:Initial *K* ⇐ [0]2:**while** TRUE **do**3: **for***i* = 1 to *N_P_***do**4:$k_{i}=\frac{F(N)}{\sum_{i}\frac{1}{P_{i}}\times P_{i}}$5: **end for**6: *counter* ⇐ 07: **for***i* = 1 to *N_P_***do**8:  **if***k_i_* > 1 **then**9:   *k_i_* ⇐ 110:   *counter* ⇐ *counter* + 111:   *P⃗* ⇐ *P⃗\P_i_*12:  **end if**13: **end for**14: **if***counter* = 0 **then**15:  **return***K*;16: **else**17:  *F*(*N*) ⇐ *F*(*N*) − *counter*18: **end if**19:**end while**

When *N* > *Q_max_*, we begin dropping *Event* packets. Furthermore, since we drop all *Non-Event* packets, *N* = *N_E_*, which means the buffer is occupied only by *Event* packets. Since the buffer utilization ratio should monotonically increase with congestion, it implies *F_E_*(*N*) ≤ *F_E_*(*N* + 1). Additionally, since the dropping probability also needs to monotonically increase with *N* to relieve congestion, we can conclude that when there are *N_E_* > *Q_max_* packets with a new incoming packet, we have *N* ≤ *F_E_*(*N*) ≤ (*N* + 1). Consequently, we have *F_E_*(*N*) = *N* + 1 − *d_E_*(*N*), where *d_E_*(*N*) is a non-decreasing function with the value bounded between [0,1]. While the above scheme looks similar to a tail-dropping scheme, it is important to point out that our mechanism is different from tail-dropping since we may drop a packet from any position in the queue. The only similarity is that the average number of remaining packets *F_E_*(*N*) is similar to that in the tail-dropping scheme. The curve *d_E_*(*N*) is shown in Figure 5, and it can also be a convex, linear or concave function. The convex function is conservative, the concave function is aggressive, and the linear function is in between since *d_E_*(*N*) is the minus term.

#### Link Quality Measurement

As we discussed in Section 2.1., collisions in MAC layer and link failures in PHY layer influence the probability that a packet is successfully received by the sink. Therefore, when the MAC layer is ready to send the packets in the queue, we also need to update *P_i_* to record the link quality information. A number of parameters can provide the link quality information, such as the number of interfering neighbors and Signal-to-Interference-and-Noise Ratio (*SINR*). However, due to limited resources in a WSN, we prefer to find an efficient parameter which can also be easily obtained through link measurement. In our system, we choose the ratio of the number of successful transmissions (*M_S_*) to the total number of transmission attempts (*M_T_*) as the metrics to indicate link quality. First, *M_T_* and *M_S_* represent the influence from both collisions in the MAC layer and transmission failures in the PHY. Second, each node can easily maintain this information by counting the transmissions in the MAC layer.

Note that wireless link quality in a WSN is usually time-variant. Therefore, recent measurement results are more important than those that are older; the new measurements can more accurately represent the current link quality. In other words, if we time 0 to *t* to be slotted into small intervals, $\frac{M_{S}(t_{2})}{M_{T}(t_{2})}$ is more valuable than $\frac{M_{S}(t_{1})}{M_{T}(t_{1})}$ as long as 0 ≤ *t*_1_ ≤ *t*_2_ ≤ *t*. On the other hand, we also do not want the instantaneous perturbation of link quality to destroy the accuracy of estimation of *P_i_*, so we cannot simply abandon the information from $\frac{M_{S}(t_{1})}{M_{T}(t_{1})}$ Therefore, we follow the basic idea of machine learning [12]. In each time interval *t_j_*, we independently collect the statistic information of $\frac{M_{S}(t_{j})}{M_{T}(t_{j})}$ and the link quality in time interval *t_j_*, denoted by *L*(*t_j_*) to be given by

(13) $$L(t_{j})=(1-\alpha )\times L(t_{j-1})+\alpha \times \frac{M_{S}(t_{j})}{M_{T}(t_{j})}$$

Then in Part *2.2* we update *P_i_* = *P_i_* × *L*(*t_j_*) where *t_j_*, is the current time. The parameter *α* will depend on the wireless network is and its value may be decided by the network administrator. If the link changes quickly, for example, as in an underwater WSN, *α* will be set close to 1 so as to incorporate more recent information. On the contrary, if the link is stable, *α* should be smaller so as to accept more history information to avoid instantaneous variation of the link.

#### Discussions

As we described in this section, different components in *PCC* realize the design considerations in Section 2.1.. First, the separate thresholds for *Event* and *Non-Event* packets in queue handler guarantee the high throughput of *Event* packets during congestion. Second, the optimization algorithm in Equations (5), (6) and (7) provides coverage fidelity of the whole network. Third, the proposed new queue dropping schemes, and corresponding packet admission probabilities *K_E_* and *K_N_* implements flexible queue scheduler. Finally, updating *P_i_* by the probability of network dropping and MAC/PHY link failure efficiently utilize the network resource.

### 2.3. Evaluation and Comparison

In this section, we evaluate *PCC* and compare its performance with other existing solutions. Compared with the dynamic queue scheduling in *PCC*, most queue schedulers, such as *FIFO* or *RED* [13] use tail-dropping. Since it is not our focus to compare different existing queue schedulers, we only show comparison results with *FIFO*. Note that the conclusions in this section still apply to other queue schedulers.

In the following simulations, we use standard IEEE 802.11 protocol for the MAC and physical layer. Ad hoc On-Demand Distance Vector (AODV) [14] is used as routing protocol in network layer and User Datagram Protocol (UDP) and Constant Bit Rate (CBR) are used in the transport and application layers, respectively. For the results presented in the following subsections, the X-axis represents the rate at which the *Non-Event* packets are generated. Sensors with events generate *Event* packets at a higher rate, which is 1.5 times the basic rate. Note that in our simulations, all the sensor nodes that detect the *Event* generate at the same rate. However, this is not a requirement of our scheme. We compare the performance based on different metrics, such as throughput, packet delay and fairness. We consider random topologies with 24 sensors and one sink, where 12 sensors are *Event* nodes and the others are *Non-Event* nodes. The following results are the average of 25 simulation runs.

#### Throughput and Delay

**Figure 6.**
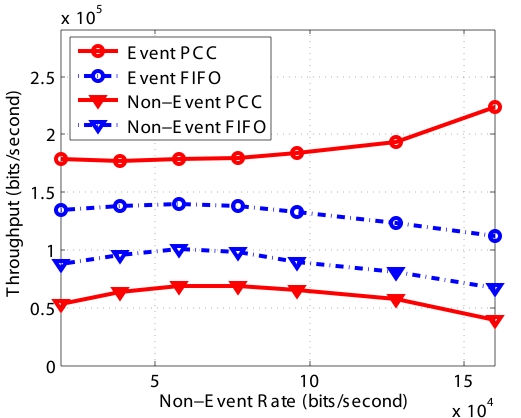
System throughput.

**Figure 7.**
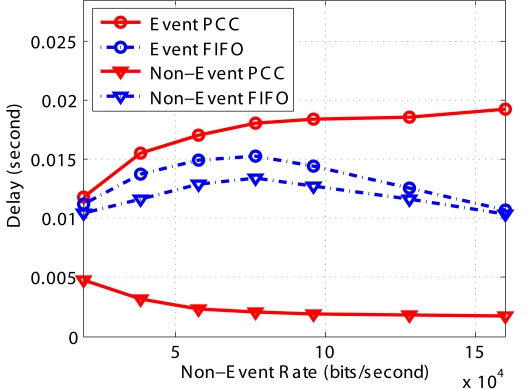
Avgerage end-to-end delay.

The throughput and end-to-end delay are shown in Figure 6 and Figure 7, respectively. In Figure 6, since *FIFO* does not distinguish *Event* and *Non-Event* packets, the capacities of “Event FIFO” and “Non-Event FIFO” are roughly proportional to the traffic generation rate. However, when the traffic generation rate exceeds the bound, which degrades the wireless link quality by introducing more collision and larger contention window in MAC protocol, the overall capacity of *FIFO* decreases. *PCC* provides *Event* packets higher priority. Therefore, the throughput of “Event PCC” keeps increasing until it reaches the whole system capacity. On the other hand, more *Non-Event* packets are dropped during congestion, and therefore the capacity of “Non-Event PCC” decreases with the increase of the basic packet generation rate. As we explained before, with the constraint of the system capacity, sinks are more interested in the *Event* packets, so the results are consistent with our design objective.

In Figure 7, it is obvious that the end-to-end delay of *Event* or *Non-Event* packets in *FIFO* almost remains the same. Since *PCC* preferentially accepts *Event* packets, *Event* packets experience longer queue delay on average. However, during congestion, only a few *Non-Event* packets reach the sink (most are dropped in the intermediate nodes) and they experience low queueing delay. Therefore, the average delay of *Non-Event* packets in *PCC* is comparably low.

#### Coverage Fidelity

**Figure 8.**
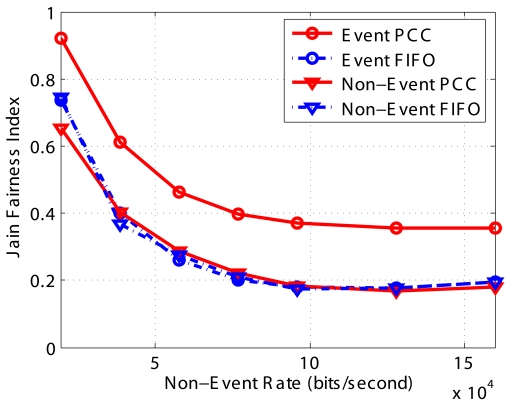
Jain's fairness of the different schemes.

The most important improvement of *PCC* is that it provides fairness to all sensor irrespective of their location, and therefore offers coverage fidelity of the whole WSN. In Figure 8, we count the number of packets from different sensors and derive the Jain's Fairness Index (*JFI*). From the results, it is clearly observed that the fairness of *FIFO* decreases with the increase in the basic packet generation rate when the network is heavily congested and only the sensors very close to the sink are able to forward their packets to the sink. However, the *JFI* of *Event* packets in *PCC* is much higher since sensors give packets equal probability to go to the next hop. Since *PCC* drops *Non-Event* packets during congestion, only a few *Non-Event* of the packets can reach the destination. Therefore, the *JFI* of *Non-Event* packets does not improve.

To explicitly compare the performance of *PCC*, we use a chain topology, which contains three sensors and one sink. Node 1 is closest to the sink; node 3 is farthest from the sink and node 2 is in the middle. The distance between the nodes are the same. All sensors are either *Event* or *Non-Event* nodes. We count the number of packets received by the sink from the three sensors, and results are shown in Table 1. We found that in *FIFO*, packets from remote nodes have a lower probability to reach the sink while in *PCC*, the network provides an equal chance to packets from all sensors. The fairness of both *Event* and *Non-Event* packets improve significantly with *PCC*.

#### *F_E_*(*N*) and *F_N_*(*N*)

**Figure 9.**
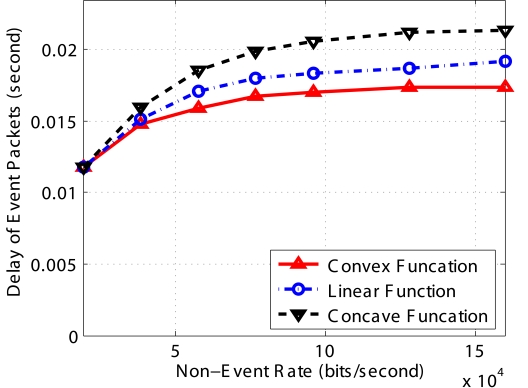
End-to-end delay for the three different functions for implementing *F_E_*(*N*)

**Figure 10.**
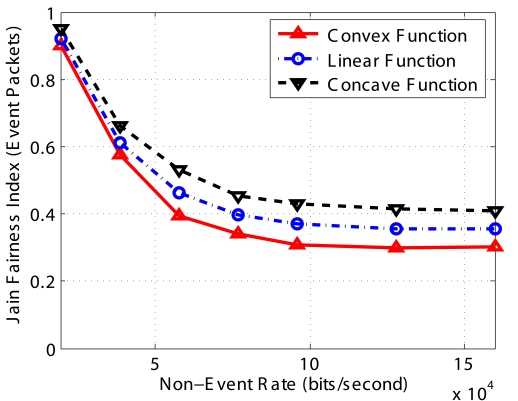
Fairness for the three different functions for implementing *F_E_*(*N*).

All the above results are based on the linear function for both *F_E_*(*N*) and *F_N_*(*N*). In this section, we compare the influence of different functions (e.g., convex, concave, direct line) on the performance of *PCC*. Since we collected the results when the network is congested and every sensor kept transmitting packets to the next hop, the throughput of the three functions are almost the same. The results of end-to-end delay and fairness are shown in Figure 9 and Figure 10. In Figure 9, the delay of the concave curves (Note that “concave” refers to *F_E_*(*N*), not *d_E_*(*N*)) is largest since this scheme conservatively kept more packets in the buffer than the other two schemes; therefore packets have longer queue delay. However, if we employ the convex function, which aggressively drop more packets, the packet delay decreases since the average queue length of all nodes is the smallest among the three schemes.

Since *PCC* can selectively drop any packets in the queue, the more packets there are in the buffer, the more options *PCC* has, which means *PCC* can provide better fairness performance and hence higher coverage fidelity. The analysis is validated in Figure 10 which shows that the performance of the concave function is the best among three schemes and that of the convex function is the worst.

#### *Q_min_* and *Q_max_*

**Figure 11.**
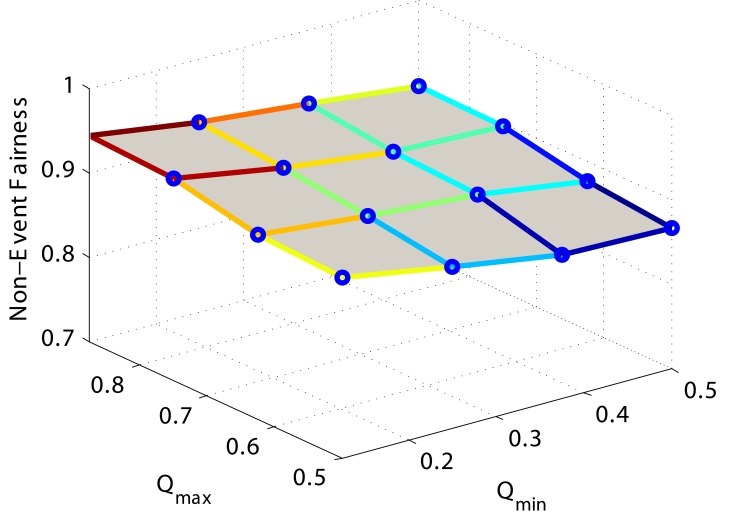
Influence of *Q_min_* and *Q_max_*.

*Q_min_* and *Q_max_* are important parameters in *PCC* since they are the thresholds in the queue scheduler to determine when *Event* and *Non-Event* packets will be dropped. Similar to previous discussion, higher values of *Q_min_* and *Q_max_* are related to higher buffer utilization, so the average packet delay is larger due to longer queueing delay. Therefore, in this section we only show the fairness results of *Non-Event* packets, which are influenced by both *Q_min_* and *Q_max_*. In Figure 11, Q*_min_* and *Q_max_* are normalized by the total buffer length. We find that when *Q_min_* is increased with fixed *Q_max_*, more *Non-Event* packets remain in the buffer without being selected by the queue scheduler. Consequently, the fairness is determined more by the wireless link quality and the lower fairness is due to the randomness. When *Q_max_* is increased with a fixed *Q_min_*, more *Non-Event* packets have the opportunity to remain in the buffer and the queue scheduler can implement the optimization algorithm to selectively drop packets. Consequently, the fairness index is higher. However, note that the influence of *Q_min_* and *Q_max_* is not obvious, or in other words, *PCC* is not very sensitive to the choice of the thresholds.

## 3. A Generalized Approach for Multiple Event Types

In general, sensor nodes may have multiple sensing devices to monitor multiple attributes of the physical environment in which they are deployed. Each of these sensing devices will generate its own *Event* and *Non-Event* packets. Consequently, simply distinguishing *Event* and *Non-Event* packets may not be enough when the WSN is in a congested state. Different sensed data will have different value to the sink and it is important to ensure that more of the valuable data is collected by the sink when the network is congested. In this section, we extend *PCC* by introducing a *Pricing System* which modifies the packet dropping policy based on different priorities of different *Event* packets to achieve a specified balance between the aggregate “value” of the collected data and coverage fidelity. The key features of the proposed *Pricing System* are the following:

- The sink acts as the information consumer and sets a price that it is willing to pay for each different types of *Event* packets. Higher prices indicates the sink prefers the sensor network to collect this corresponding category of *Event* packet at the cost of more transmission resource. The ratio of different prices determines the balance between the priority and coverage. If all prices are equal, the *Pricing system* degrades to *PCC*. If one of the prices is ∞, the sink is willing to only accept the corresponding category of *Event* packets and consequently the wireless sensor network would block all other types of *Event* packets.
- The sensors operate as the information providers and when congested selectively drop packets according to the value that the sink places on the information in each packet (determined by the price set by the sink). When the buffer utilization is high, the sensor tends to keep packets with the lower accumulated survival probability *P_i_* and higher price. The detailed algorithm is introduced in Section 3.1..
- The prices can dynamically vary according to the changes in the physical environment and the network condition. When the sink modifies the prices, the new prices are broadcast to the entire network and each sensor node uses the new prices to adjust the dropping policy during congestion.

The *Pricing System* gives more flexibility to the network administrators. It is easy to add or delete a category of information by adding a new price or setting the price to zero, as long as the hardware can sense the corresponding type of information. Adjusting the ranking of different types of *Event* packets can also be done through changing the prices of the data collected by the sensors. In addition, the prices configured by the network administrators is able to accurately control the dropping probabilities, and thus control the ratio of received packets in the sink. Based on the structure of *PCC*, we describe the algorithm for the *Pricing System* in Section 3.1. and evaluate the performance improvement in Section 3.2..

### 3.1. Protocol and Algorithm

The structure of the *Pricing System* is almost the same as *PCC* described in Section 2.1.. However, in order to support multiple types, it is necessary to modify the communication protocol and the dropping strategy as described below.

#### Task 1

In the *Pricing System*, the sink needs to broadcast the updated prices to all sensors in the network. This functionality could be implemented on multiple layers, such as application, network or MAC layers. In order to avoid additional burden to the network, the *Pricing System* broadcasts the prices through the *ACKs* of the MAC layer so as not to introduce a new protocol. In wireless networks, *ACKs* in the MAC layer is inevitable due to the *CSMA/CA* protocol as the sender needs the confirmation of the transmission from the receivers. In the proposed approach, the sink could update the prices and notify the nodes within one hop when it receives their data frames. Later those sensors receiving the new prices could propagate the information to their neighbors. This process will eventually ensure that all sensors are aware of the new prices. This process does take some time to propagate the updated information to the whole network. However, note that the MAC layer transmissions occur frequently even without any data communication. For example, most routing protocols need to detect whether the next hop is still alive, which triggers periodic transmissions between two neighbors at the MAC layer.

In order to support the above approach, it is also necessary to modify the format of *ACK* frame. Suppose there are totally *M* types of packets, *M* − 1 types of *Event* packets and one type of *Non-Event* packet. In the payload of an *ACK*, *M* variables (2 bytes for each) present the prices of all categories; and one variable presents the *time stamp*, with which the nodes can compare the newly received prices with the stored ones. Therefore, the possible price range is 2^16^ ≈ 64*K*; and the length of the time stamp is 64*K*, which could be utilized circularly if necessary.

#### Task 2

Unlike *PCC*, *Pricing System* supports multiple *Event* types. Therefore, in the header of each packet, we augment an additional part with *n* = *log*_2_*M* bits to label the type of the packet. When a sensor generates a packet, it sets the header with the category so that all nodes along the path to the sink are able to process this packet according to the dropping strategy introduced below.

The overall structure of the algorithm is similar to Figure 4, except that we replace the Part *2.1* with the following new dropping strategy. To support the multiple categories of events, we introduce a new notation *R_i_*, which is the *price* of packet *i. R_i_* can be any one of the *M* prices, ranging from 1 to 64*K*. With the definition of *R_i_*, the Part *2.1* becomes

1. 0 ≤ *N* ≤ *Q_min_*: Keep all packets since the utilization of the buffer is low.
2. *Q_min_* ≤ *N* ≤ *Q_max_*: Keep all types of *Event* packets and begin to drop *Non-Event* packets according to the function *F_N_*(*N*) shown in left part of Figure 5. The optimization problems becomes
  (14) $${\text{maximize}}_{\left\{{\vec{K}}_{N}\right\}}\frac{{\left(\sum_{i\in N_{N}}\frac{P_{i}\times k_{i}}{R}\right)}^{2}}{N_{N}\times \sum_{i\in N_{N}}{\left(\frac{P_{i}\times k_{i}}{R}\right)}^{2}}$$
  such that
  (15) $$\sum_{i\in N_{N}}k_{i}=F_{N}(N)$$
  (16) $$\forall i\in N_{N}k_{i}\in [0,1]$$
  where *R* is the price of *Non-Event* packets, and *K⃗_N_* = [*k*_1_, *k*_2_, …, *k_NN_*] is the accepting probability, which is the decision variable. In the optimization algorithm, we would like the ratio of different type of price to be equal to the ratio of the cumulative survival probability of different types of packets as much as possible. The ideal case is when the Jain's Fairness Index equals 1, which is achieved when *R*_1_ : *R*_2_ : … : *R_M_* = *P*_1_*k*_1_ : *P_2_k_2_* : … : *P_M_k_M_*. In other words, we ensure that *Event* packets for which the sink is willing to pay a higher price has higher accumulated survival probability (*P_i_k_i_*) and the ratio of the cumulative survival probability follows the ratio of the prices. If two classes of packets traverse through similar network conditions, the ratio of throughput of these two types of packets should be similar to the ratio of the prices. Note that network condition includes both network link quality and the probability of being dropped in a node along the path to the sink. If the prices of two classes of packets are the same, we would like the probability of packets received at the sink to be the same. If all the prices are equal, the optimization problem becomes the same as Section 2.. Since all *Non-Event* packets have the same price and we selectively drop *Non-Event* packets, Equation 14 becomes
  (17) $${\text{maximize}}_{\}{\vec{K}}_{N}\{}\frac{{\left(\sum P_{i}\times k_{i}\right)}^{2}}{N_{N}\times \sum {\left(P_{i}\times k_{i}\right)}^{2}}$$
  Note that if *R* = 0, then *k_i_* = 0.
3. *Q_max_* ≤ *N* ≤ *Q*: After dropping all *Non-Event* packets, begin dropping *Event* packets since the buffer is highly utilized. The dropping strategy follows the optimization problem given by,
  (18) $${\text{maximize}}_{\left\{{\vec{K}}_{E}\right\}}\frac{{\left(\sum_{i\in N_{E}}\frac{P_{j}\times k_{j}}{R_{j}}\right)}^{2}}{N_{E}\times \sum_{i\in N_{E}}{\left(\frac{P_{j}\times k_{j}}{R_{j}}\right)}^{2}}$$
  such that
  (19) $$\sum_{j\in N_{E}}k_{j}=F_{E}(N)$$
  (20) $$\forall j\in N_{E}k_{j}\in [0,1]$$
  where *R_j_* is the price of packet *j*, and *K⃗_E_* = [*k*_1_, *k*_2_, …, *k_NE_*] is the accepting probability, which is the decision variable. The meaning of the optimization is the same as explained in last paragraph. *F_E_*(*N*) = *N* + 1 − *d_E_*(*N*) and the *d_E_*(*N*) function are shown in the right part of Figure 5. Note that, if *R_j_* = 0, then *k_j_* = 0.

The algorithm is similar to Algorithm 1. The only difference is to set *P_i_/R_j_* instead of *P_i_*. Furthermore, the computation complexity is the same as *PCC*, which is *O*(*N*^2^).

### 3.2. Simulations

In this section, we compare the performance of our *Pricing System* with FIFO. Note that the results shown here also apply to other tail dropping active queue management algorithm such as RED. We consider total throughput, throughput per class, fairness and “value” as the performance metrics for the performance comparison. In a multihop wireless network, $\sum_{i=1}^{N}h_{i}$ indicates the capacity of network, where *N* is the number of packets successfully received at the destination node, and *h_i_* is the number of hops traversed by packet *i* from source to destination. The implicit assumption is that all packets are equally important. In this study, we consider a WSN with several classes of packets with different priorities. We use price *R_i_* in our *Pricing System* to indicate the relative priority of packet *i*. Based on this, we define the new metric “value” as $\sum_{i=1}^{N}R_{i}*h_{i}$, where *R_i_* is the price of packet *i* and *h_i_* is the number of hops traversed by packet *i* from source to destination. The higher the “value”, the more information is collected from the WSN.

We evaluate the correctness and performance of our algorithm using a chain topology and a random topology. The chain topology is used as a base case to analyze and validate the results. In the simulations, IEEE 802.11 is used at the MAC/PHY layer, AODV is used as the routing protocol in the network layer, UDP is set as the transport layer protocol and CBR traffic source is used in the application layer.

**Figure 12.**
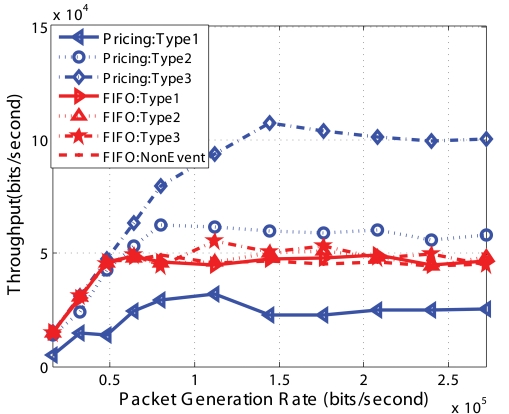
Throughput of multiple types of packets for chain topology.

The chain scenario consists of five nodes in a linear topology with equal distance between nodes. Node 1 is the sink and all packets generated by node 5 pass through nodes 4, 3, 2 to reach node 1. Node 5 generate *Non-Event* packets and three types of *Event* packets with price 2, 4, and 8 units, respectively. In order to explicitly evaluate the performance of our algorithm, we set *Q_min_* and *Q_max_* to 0 so that our optimization algorithm is always active during the simulation. Results are shown in Figure 12 to Figure 15. The x-axis in all the figures is packet generation rate which is set to be the same for all the four (*Non-Event* and three *Event*) types of packets.

**Figure 13.**
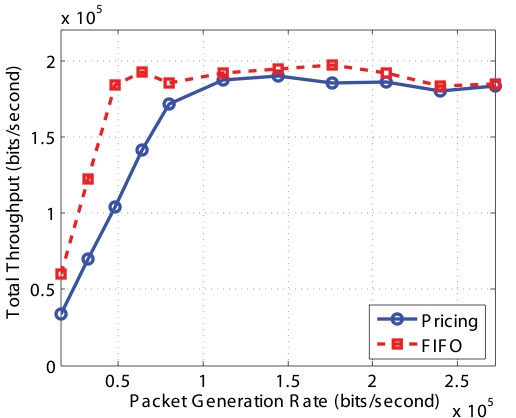
Aggregate system throughput for chain topology.

From Figure 12 we can see that for FIFO which does not differentiate the packets, the throughput of different *Event* packets and *Non-Event* packets are almost the same. For our *Pricing System*, type 3 *Event* packets have the highest throughput since they have the highest price; type 1 *Event* packets have the lowest throughput since they have one fourth of type 1 price and one half of type 2 price. No *Non-Event* packets are received by the sink since they are all dropped. We set *Q_max_* equal to 0 as to explicitly test our optimization algorithm, therefore, all *Non-Event* packets are dropped. We also find that the throughput of the type 1 *Event* in the Pricing System is less than that of FIFO. Since the total throughput of the network is fixed, the increased throughput of type 3 decrease the throughput of type 1. The total throughput is shown in Figure 13.

From Figure 13, we can see that the throughput increases with packet generation rate until the network capacity is reached at which point it saturates. We can also see that the total throughput of FIFO and our *Pricing System* has the same trend. However, the total throughput of our Pricing System is lower than that of FIFO because both *Q_min_* and *Q_max_* are 0. Consequently, the queue utilization is lower. But the total throughput should be almost the same for FIFO and Pricing System, which can be seen from our random topology simulation where *Q_min_* = 1/3 * *QueueSize* and *Q_max_* = 2/3 * *QueueSize*.

**Figure 14.**
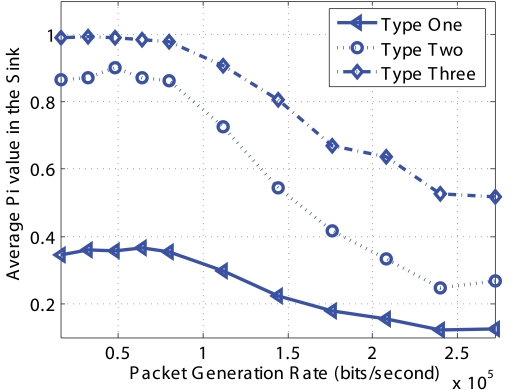
Pi values of different types of *Event* packets for chain topology.

To validate the design of our Pricing System, Figure 14 shows the the average *P_i_* values of the received packets at the sink. Note that the *P_i_* value in the sink means the successful transmission probability of packet to the sink. When the packet generation rate is small and there is sufficient network capacity, the successful transmission probability is higher. When the packet generation rate is high and the network becomes congested, the probability of successful transmission becomes smaller. The most important validation here is that when the network is highly congested, the ratio of average *P_i_* values is almost the same as the ratio of price. For example, when the packet generation rate is 272 kbps, *P*_1_ = 0.1253, *P*_2_ = 0.2686, and *P*_3_ = 0.5176, which is in the same ratio as the price for the different types of packets namely, 2 : 4 : 8.

**Figure 15.**
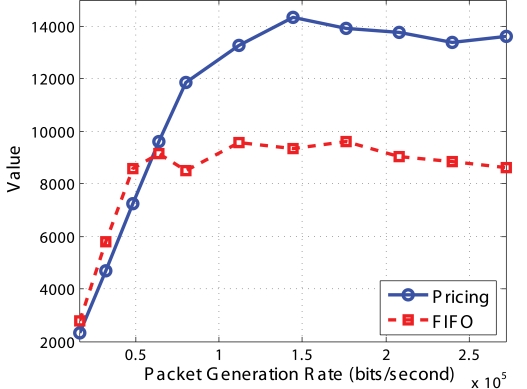
Comparison of value for chain topology.

Figure 15 plots the “value” as a function of the packet generation rate. We see that the “value” of Pricing System is much better than FIFO when network is congested. When the traffic generation rate is low, the “value” is smaller than FIFO due to the low utilization of the queue buffer. It is not the case when *Q_min_* = 1/3 * *QueueSize* and *Q_max_* = 2/3 * *QueueSize*, which will be shown in the random topology simulation.

**Figure 16.**
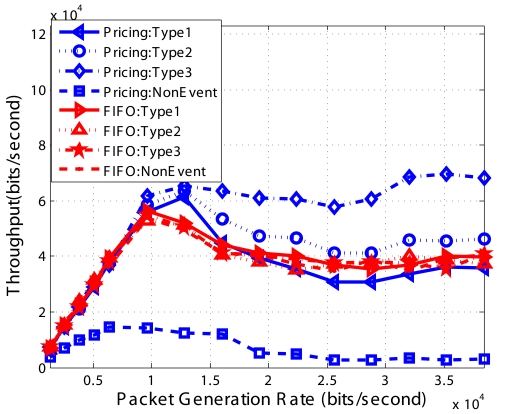
Throughput of different *Event* types for random topology.

Figure 16 to Figure 19 show the simulation results for random topologies; the results are average of 25 simulation experiments which corresponds to 25 different random topologies. Each random topology contains 25 nodes, including a sink. Eight of the nodes send *Non-Event* packets and 3 types of *Event* packets with the price 2 : 4 : 8. Other nodes do not generate packets but forward packets to the sink. The X-axis in the figures is the packet generation rate which is the same for each of the different types. In these simulations, we set *Q_min_* = 1/3 * *QueueSize* and *Q_max_* = 2/3 * *QueueSize* and the dropping functions are linear functions shown in Figure 5.

Figure 16 shows the throughput of different types of packets using FIFO and our *Pricing System*. First, the throughput of different types packets using FIFO are almost the same, since FIFO does not differentiate different type of packets. Second, when the network is not or lightly congested, FIFO and Pricing System has the similar throughput. But the throughput of *Non-Event* packets using *Pricing System* is smaller than that using FIFO, because the *Pricing System* begins to selectively drop some *Non-Event* packets so as to avoid congestion. Third, when network is highly congested, the throughput of type 3 and type 2 packets using Pricing System are much higher than those using FIFO. The *Pricing System* is able to guarantee higher probability of successful transmission of packets with higher priority when network is congested. Furthermore, the ratio of successful transmission of packets is consistent with the ratio of price decided by the network operator.

**Figure 17.**
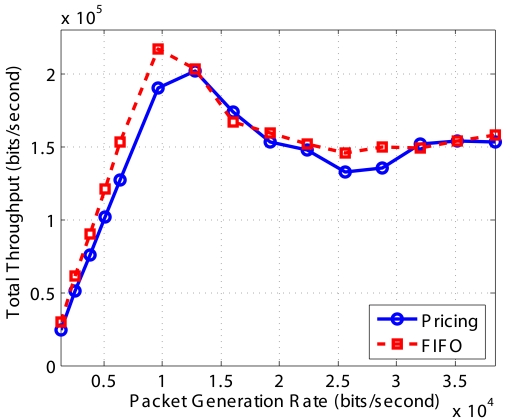
Aggregate system throughput for random topology.

Figure 17 shows the total throughput of all types of packets using FIFO and our Pricing System. We can see that their throughputs are similar. In a wireless network, throughput increases as packet generation rate increasing. When the network is saturated, the total throughput decreases lightly because of the severe MAC layer contention. The FIFO line is more smooth since FIFO only drop packets when the buffer size is full. However, the pricing line has some randomness, since our *Pricing System* drop packet using the probability obtained from our optimization algorithm.

**Figure 18.**
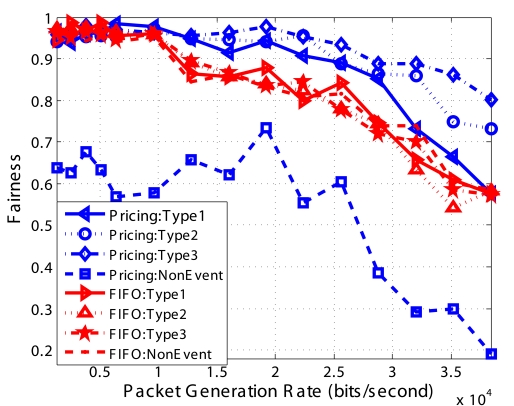
Fairness of different classes of *Event* packets for random topology.

We show Jain's Fairness Index of different types of packets in Figure 18. Our optimization algorithm lets packets with the same price have the same probability of success to reach the sink. Our simulation results show that our *Pricing System* has higher fairness than FIFO. But the fairness of *Non-Event* packets using *Pricing System* has lower fairness than FIFO. This is because we drop all *Non-Event* packets when buffer size is bigger than *Q_max_*.

**Figure 19.**
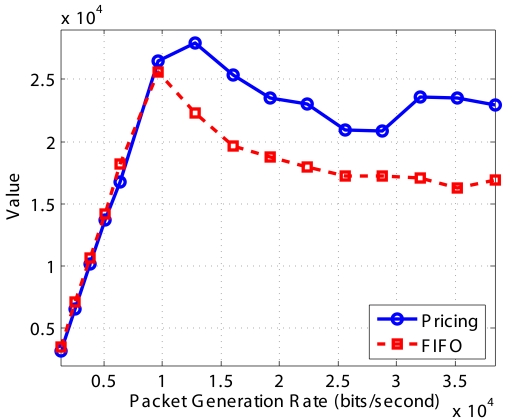
Comparison of value for random topology.

Figure 19 shows the simulation results of our newly defined metric “value”. When network is not congested, the values of FIFO and *Pricing System* are almost the same. However, when the network is congested, the proposed *Pricing System* receives more packets with higher priority and has much higher value than FIFO.

## 4. Related Work

Prior works on congestion control mechanisms in WSNs are mainly embedded in the end-to-end controls, such as CODA [15], ESRT [7], STCP [16], PORT [17], SenTCP [18] and [19]. The underlying method in these papers is the use of end-to-end rate adjustment to fulfil congestion control. These protocols detect and prevent congestion by reducing the number of packet retransmissions and energy used. We briefly summarize the main contributions of these papers. Congestion Detection and Avoidance(CODA) is one of the early papers discussing congestion control in wireless sensor networks. CODA is a energy efficient scheme which comprises of three mechanisms: (1) receiver-based congestion detection, (2) open-loop hop-by-hop backpressure, and (3) closed-loop multi-source regulation. CODA is evaluated by two metrics proposed by the authors, namely, energy tax and fidelity penalty. Event-to-Sink Reliable Transport (ESRT) is based on the observation that sensor networks are event-based systems. ESRT protocol operation is determined by the network state in terms of congestion condition in the network and path reliability. Simulation analysis of ESRT shows that proposed transport protocol achieves desired reliability with minimum energy consumption. Sensor Transmission control Protocol (STCP) is a scalable and reliable transport layer protocol for sensor networks. STCP is central control protocol since most of the functionalities are implemented at the base station. Simulations show that STCP can increase network lifetime and achieve high reliability. Price-Oriented reliable Transport (PORT) protocol is proposed to obtain reliability and minimize energy consumption. Price refers to the communication cost between sources and the sink. PORT uses price information to achieve reliability. Minimization of energy consumption is achieved by two schemes, upstream information optimization of the sink and downstream optimal routing scheme locally implemented in sensor nodes. Simulations show the effectiveness of PORT for reducing energy consumption comparing to existing schemes. SenTCP is an open-loop hop-by-hop congestion control protocol for wireless sensor networks to improve system throughput, reduce packet dropping, and minimize energy consumption. The work in [19] proposes a congestion control using the congestion degree calculated by the remaining buffer size and net flow.

Rate-Controlled Reliable Transport (RCRT) protocol proposed in [20] ensures efficient and flexible rate control like previous protocols. However, RCRT has the improvement that combine reliable transmission and congestion control together. Congestion detection and rate adaptation functionality are performed by the sink. The author also evaluated RCRT on a 40-node wireless sensor network testbed and show that it achieves better performance compared with IFRC [8].

The studies reported in [21–24] address the congestion problem using routing protocols. In [21], congestion control is achieved by dividing the monitoring areas into several subareas and adjust the local and forwarding traffic based on the transmission parameter. In [22], an interference-minimized multipath routing protocol is proposed for load balancing and a congestion control scheme to reduce the loading rate of the source. The main idea of [23] is to find a less congested node to forward packets to when congestion occurs. In [24] a routing protocol is proposed for congestion control in WSNs by selecting a route which use Network Allocation Vector (NAV)[25] information to determine the channel status. Our optimization algorithm is orthogonal with these protocols since they work in different layers.

Other research based on priority fairness are [26], IFRC [8], Fusion [27], and [28–30]. The study reported in [26] gives a design of a distributed, scalable congestion elimination mechanism in the transport layer, which ensures fair delivery of packets to the sink when using either a probabilistic selection or a epoch-based proportional selection. Interference-Aware Fair Rate Control (IFRC) discusses a mechanism for each node to detect the contending flows locally and fairly by adapting its own transmission rate and using a congestion sharing mechanism. It can achieve MAC layer fairness, but not application layer fairness. Application layer fairness is more important to users. Fusion combines three mechanisms that span to different layers. They are hop-by-hop flow control, rate limiting source traffic, and a prioritized MAC protocol. Hop-by-hop flow control is used for congestion detection and mitigation. Rate limiting is used to prevent unfairness toward sources which are far from the sink. A prioritized MAC scheme is designed for congested nodes to have higher priority to access the channel as to quickly drain out their buffer. The works reported in [28–30] share a similar idea and use node priority index to reflect the importance of each node for priority-based congestion control. These papers neglect the details of MAC protocols and assume they provide even access opportunities for each node, which neglect the important characteristic of time-varying wireless links in WSNs. Finally, the priority index design is based on node priority, not priority of different classes of information.

To the best of our knowledge, there is only one paper that discusses congestion control for heterogeneous traffic [31]. But the protocol did not consider the wireless link characteristic, fairness and coverage fidelity. Our scheme, however, can efficiently collect different categories of information based on their relative priorities and also consider the affect of wireless links to achieve fairness.

## 5. Conclusion

In this paper, we have proposed a new scheme *PCC* to address congestion problem in a WSN and then we extend *PCC* to efficiently collect multiple categories of information in an advanced WSN. In *PCC*, we assign different priorities to *Event* and *Non-Event* packets, which have different values in a WSN. We propose an optimization algorithm to provide fair opportunity to sensors irrespective of their locations. We present a novel queue scheduler, which can drop any packets in the queue, supplies much more flexibility to information collection during congestion. Finally, also carefully involve the factor of different wireless link qualities and utilize the statistic information to adjust the dropping decision. In *PCC*, sensors only need to collect local information about the queue in the network layer and link quality in MAC layer, which is scalable and practical for large WSNs. Our analysis and simulation show that *PCC* can achieve high *Event* throughput and much better fairness and hence higher coverage fidelity. We also discussed the influence of some of the parameters on *PCC*, such as admission function and two thresholds for *Event* and *Non-Event* packets.

In the *Pricing System*, we propose an optimization algorithm for the queue scheduler. The *Pricing System* is simple and efficient to distribute network resources to different *Event* packet according to the decision of the network operator. The *Pricing System* is carried out when the network is congested. Following the design, we can control congestion and fully utilize the WSN. Our simulations show that higher throughput can be achieved for packets with higher price, and fairness can be guaranteed within one category.

## Figures and Tables

**Table 1.** Packets successfully received from different sensors in a chain topology.

	Node 1	Node 2	Node 3	Jain's Fairness Index
*Event PCC*	139	130	135	0.99925
*Event FIFO*	297	77	20	0.54735
*Non-Event PCC*	149	125	124	0.99247
*Non-Event FIFO*	292	60	23	0.52437

## References

[1] Paek J., Chintalapudi K., Govindan R., Caffrey J., Masri S. (2005). A Wireless Sensor Network for Structural Health Monitoring: Performance and experience.

[2] Rahimi M., Baer R., Warrior J., Estrin D., Srivastava M.B. (2005). In Situ Image Sensing and Interpretation in Wireless Sensor Networks.

[3] Qiu X., Ghosal D., Mukherjee B., Yick J., Li D. (2008). Priority-Based Coverage-Aware Congestion Control for Multihop Sensor Networks.

[4] Wikipedia. http://en.wikipedia.org/wiki/Motes.

[5] Dutta P., Taneja J., Jeong J., Jiang X., Culler D. (2008). A Building Block Approach to Sensornet Systems.

[6] Cheng X., Mohapatra P., Lee S., Banerjee S. (2008). Performance Evaluation of Video Streaming in Multihop Wireless Mesh Networks.

[7] Sankarasubramaniam Y., Akan O.B., Akyilidiz I.F. (2003). ESRT: Event-to-Sink Reliable Transport in Wireless Sensor Networks.

[8] Rangwala S., Gummadi R., Govindan R., Psounis K. (2006). Interference-Aware Fair Rate Control in Wireless Sensor Networks.

[9] Akyildiz I.F., Su W., Sankarasubramaniam Y., Cayirci E. (2002). A Survey on Sensor Networks. IEEE Commun. Mag..

[10] Yick J. (2006). Advanced Services in Wireless Sensor Networks. Ph.D Thesis.

[11] Jain R. (1991). The Art of computer systems Performance Analysis.

[12] Mitchell M.T. (1997). Machine Learning.

[13] Floyd S., Jacobson V. (1993). Random Early Detection Gateways for Congestion Avoidance. IEEE ACM Trans. Netw..

[14] Perkins C., Royer E. (1999). Ad-hoc On-Demand Distance Vector Routing.

[15] Wan C.-Y., Eisenman S.B., Campbell A.T. (2003). CODA: Congestion Detection and Avoidance in Sensor Networks.

[16] Iyer Y.G., Gandham S., Venkatesan S. (2005). STCP: A Generic Transport Layer Protocol for Wireless Sensor Networks.

[17] Zhou Y., Lyu M.R. (2005). PORT: A Price-Oriented Reliable Transport Protocol for Wireless Sensor Networks.

[18] Wang C., Sohraby K., Li B. (2005). SenTCP: A Hop-by-Hop Congestion Control Protocol for Wireless Sensor Networks.

[19] Sheu J.-P., Hu W.-K. (2008). Hybrid Congestion Control Protocol in Wireless Sensor Networks.

[20] Paek J., Govindan R. (2007). RCRT: Rate-Controlled Reliable Transport for Wireless Sensor Networks.

[21] Liu Y., Liu Y., Pu J., Xiong Z. (2008). A Robust Routing Algorithm with Fair Congestion Control in Wireless Sensor Network.

[22] Teo J.-Y., Ha Y., Tham C.-K. (2008). Interference-Minimized Multipath Routing with Congestion Control in Wireless Sensor Network for High-Rate Streaming. IEEE Trans. Mobile Comput..

[23] Chen J., Zhou M., Li D., Sun T. (2008). A Priority Based Dynamic Adaptive Routing Protocol for Wireless Sensor Networks.

[24] Hsu Y.-P., Feng K.-T. (2008). Cross-layer Routing for Congestion Control in Wireless Sensor Networks.

[25] Society I.C. (1997). IEEE Standard 802.11: Wireless LAN Medium Access Control (MAC) and Physical Layer (PHY) Specifications..

[26] Ee C.T., Bajcsy R. (2004). Congestion Control and Fairness for Many-to-One Routing in Sensor Networks.

[27] Hull B., Jamieson K., Balakrishnan H. (2004). Mitigating Congestion in Wireless Sensor Networks.

[28] Wang C., Sohraby K., Lawrence V., Li B., Hu Y. (2006). Priority-based Congestion Control in Wireless Sensor Networks.

[29] Li Z., Liu P.X. (2007). Priority-based Congestion Control in Multi-path and Multi-hop Wireless Sensor Networks.

[30] Yaghmaee M.H., Adjeroh D. (2008). A New Priority based Congestion Control Protocol for Wireless Multimedia Sensor Networks.

[31] Monowar M.M., Rahman M.O., Hong C.S. (2008). Multipath Congestion Control for Heterogeneous Traffic in Wireless Sensor Network.

